# Frosted branch angiitis presenting after a SARS-CoV-2 infection

**DOI:** 10.1186/s12348-022-00316-z

**Published:** 2022-11-10

**Authors:** Akhila Alapati, Nathaniel Cameron, Sean Gratton, Erin Stahl, Mary Champion

**Affiliations:** 1 University of Kansas Medical Center, Department of Ophthalmology, 7400 State Line Road, Prairie Village, KS 66208 USA; 2 Children’s Mercy Hospital, Kansas City Missouri Department of Pediatric Ophthalmology, 2401 Gillham Road, Kansas City, MO 64108 USA; 3 University of Missouri-Kansas City Departments of Neurology and Ophthalmology, 2464 Charlotte Street, Kansas City, MO 64108 USA

**Keywords:** COVID-19, FROSTED-branch, Vasculitis

## Abstract

**Purpose:**

To report a case of frosted branch angiitis presenting in a pediatric patient with unremarkable laboratory work-up apart from SARS-CoV-2 IgG antibodies.

**Observations:**

Less than four weeks after a SARS-CoV-2 infection, a 10 year-old female presented to the emergency department with severe headache and intermittent fevers. During her hospital admission, the ophthalmology service was consulted for blurry vision. Subsequent eye examination revealed frosted branch angiitis. The patient initially received intravenous corticosteroids but was escalated to plasmapheresis to achieve resolution of her symptoms. Outpatient maintenance therapy consisted of an oral Prednisone taper and Infliximab infusion.

**Conclusion and importance:**

This case represents a unique ocular manifestation of COVID-19, as recent SARS-CoV-2 was the sole identifiable cause of the patient’s frosted branch angiitis. Additionally, this patient required plasmapheresis to control disease progression.

## Introduction

Frosted branch angiitis is a descriptive term for a retinal vasculitis that presents with significant lymphoplasmacytic infiltration of the perivascular space. Clinically, this appears like frosted branches of a tree [1].

Patients are usually affected in a bimodal age distribution, one peak in early childhood and another into the second or third decade of life, with a predominance of females to males. Frosted branch angiitis can present as an idiopathic disorder or secondary to a systemic condition. It has been associated with sarcoidosis, syphilis, tuberculosis [2], multiple sclerosis, systemic lupus erythematosus [3], pars planitis, tuberculous retinal vasculitis, cytomegalovirus [4] (CMV), herpes simplex virus (HSV), herpes zoster virus (HZV), acquired immunodeficiency syndrome (AIDS), toxoplasmosis, Bechet’s [5], Crohn’s disease, malignancy [6], and paraneoplastic syndromes [7].

In cases of infectious etiologies, most notably CMV, frosted branch angiitis can be the result of direct infection of retinal vasculature [8, 9]. When direct involvement is ruled out, frosted branch angiitis is thought to be the result of hypersensitivity-mediated deposition of immune complexes in the setting of infectious and autoimmune etiologies described above [8]. In up to 33% of cases, there is no confirmed etiology apart from a presumed viral prodrome, with these cases classified by Kleiner as “acute idiopathic” FBA [1, 8].

A targeted work up is appropriate if a patient presents with a known underlying condition. Otherwise, an ocular presentation may be the first symptom of an underlying systemic condition. An extensive work up begins with a thorough ophthalmic exam including indirect fundus examination, optical coherence tomography of the macula and nerve, and fluorescein angiography. Visual Field and an electroretinography could help aid in diagnosis and prognostic assessment. Infectious workup, such as viral titers, may be beneficial if demonstrated to be negative. Laboratory and imaging studies for both infectious and auto-immune etiologies are summarized in Table 1 below. If there are accompanying neurological signs, investigation with lumbar puncture and MRI imaging may be considered.

**Table 1:** Introductory imaging and laboratory studies for frosted branch angiitis

Infectious Investigation	
Complete Blood Count with Differential	
Viral titers for CMW, HSV, VZV	
Supplemental Infectious Investigation	
Chest X-Ray	
PPD or TB Quantiferon	
Syphilis IgG Testing	
Autoimmune- Mediated Investigation	
Chest X-Ray	
ACE, Lysozyme	
ESR & CRP	
Antinuclear Antibody (ANA), dsDNA Antibody	
Neurological Involvement Investigation	
Lumbar Puncture	
MRI Head & Orbit	

Most cases of frosted angiitis reported between 1976 and 2003 have been treated with systemic steroids and/or acyclovir in cases of herpetic etiology and systemic steroids in autoimmune or idiopathic etiologies. These patients mostly had rapid resolution and recovery of vision. A total of 10% of these reported cases have a final visual acuity of 6/60 or worse. Complications include an epiretinal membrane, retinal fibrosis, and atrophic lesions [8]. Finally, recurrence of the disease is typically rare, though there has been a reported case of secondary frosted branch angiitis due to toxoplasmosis that recurred twice after initial treatment [10].

## Case report

**Fig. 1:**
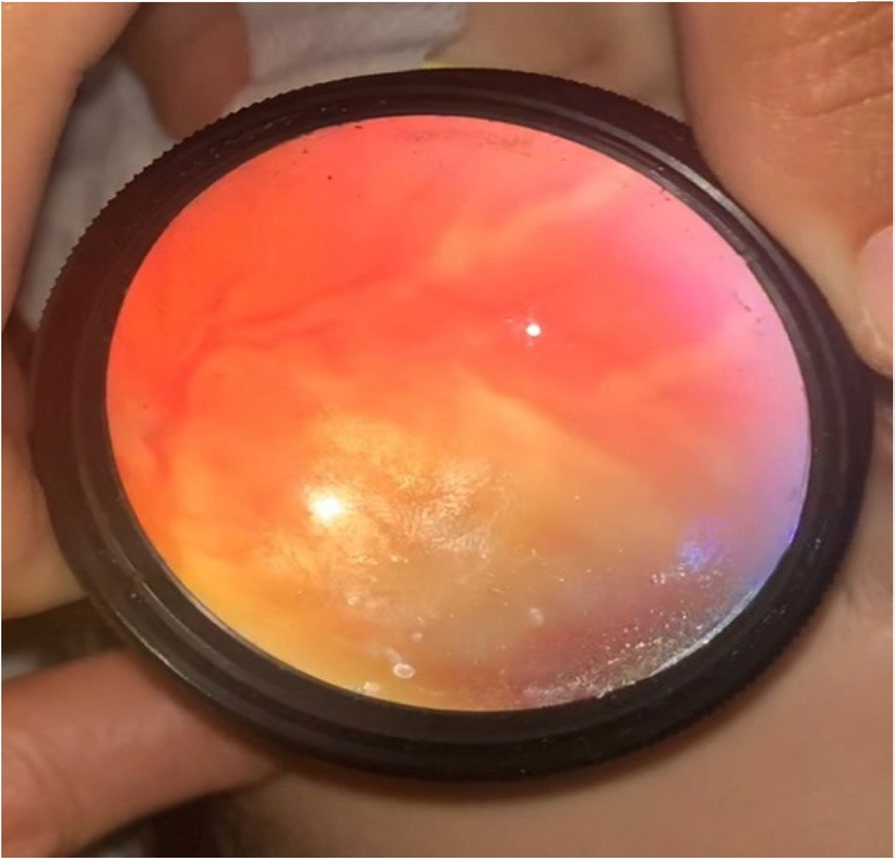
Bedside indirect Ophthalmoscopy of the left eye showing exudative retinal detachment. Image has been inverted to focus on the superior arcade

**Fig. 2:**
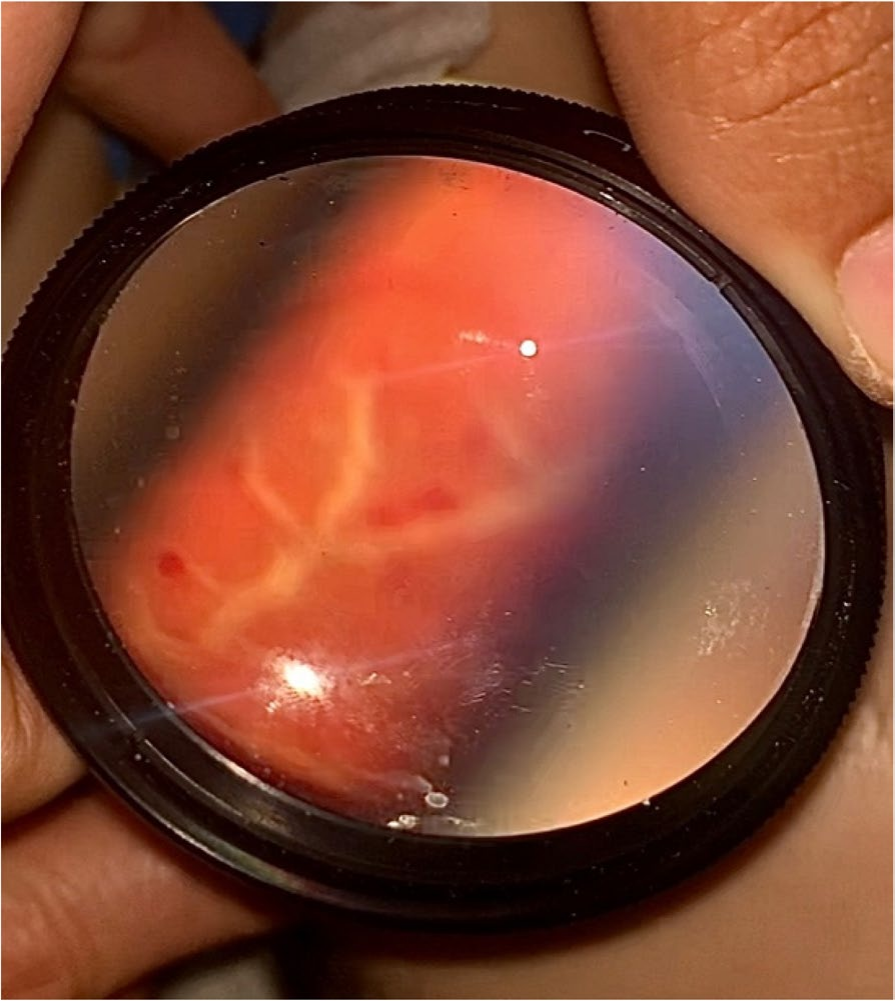
Bedside indirect ophthalmoscopy of the left eye showing perivascular sheathing consistent with “frosted branch” angiitis, and retinal hemorrhages. Image has been inverted to focus on the superior arcade

A 10 -year-old female presented to the emergency department with a persistent severe headache, intermittent fevers and a SARS-CoV-2 infection less than four weeks prior. (This presentation was prior to the availability of COVID-19 vaccinations.) A few days into her hospital stay, the ophthalmology service was consulted for blurry vision. On bedside examination, her near visual acuity was 20/400 in each eye, with mild vitreous inflammation noted on slit lamp examination. Fundoscopic examination of both eyes demonstrated optic disc and macular edema, intra-retinal hemorrhages, and peripheral vascular sheathing consistent with frosted angiitis (Figs. 1 and 2).

On neurological examination, pertinent positives included altered mental status and an afferent pupillary defect (APD). However, the APD was noted following her serous detachment. The patient demonstrated normal reflexes with no focal weakness or altered muscle tone.

Comprehensive laboratory work up demonstrated the following pertinent positives: lumbar puncture with an elevated intracranial pressure (32 cm^3^, Normal 7.5-20 cm^3^), elevated D-dimer (0.71 mcg/mL, Normal < 0.50), elevated ACE (3.2 unit/L) and positive SARS COV2 IgG and negative IgM antibodies. Testing for underlying rheumatological, viral, fungal, and bacterial etiologies are summarized in Table 2. Anterior Chamber (AC) tap was deferred due to negative infectious work-up and completion of a lumbar puncture. Given the consideration for a post-viral inflammatory consideration, neuro-imaging was obtained. This demonstrated concerns for increased T2 hyperintensity in the bilateral medial temporal lobes but was otherwise negative for evidence of cerebral vasculitis, infection, or demyelination.

**Table 2:** Summary of laboratory testing and results

Bacterial Studies	
Blood Culture No growth	
Bartonella Antibody Negative	
TP Quantiferon Negative	
Viral Testing	
SARS COV2 IgG Positive, IgM Negative	
CVM Negative (Quantitative PCR plasma)	
HSV Negative (Quantitative PCR plasma)	
VZV Negative (Quantitative PCR plasma)	
Autoimmune workup	
ESR normal	
ANA negative	
CRP 0.5 (normal)	
HLA-B51 Negative	
Myeloperoxidase Antibody Negative	
Ace 3.2 unit/L	
Neurological Studies	
CSF Bacterial Culture and Gram Stain Negative	
CSF CMV PCR and HSV PCR Negative	
CSF Fungal Culture Negative	
LP Opening Pressure 32 cm3	

Her visual status declined to hand motion when she developed a serous retinal detachment in the left eye. Intravenous corticosteroids (15 mg Methylprednisolone q6 hours for ten days) were initiated with no improvement. Seven days of IV corticosteroids yielded no improvement, with persistent headaches. Because of the patient’s lack of improvement in vision and systemic symptoms (headaches), plasmapheresis was initiated at 44 mL/kg with 1.1 plasma volume exchanged for a duration of 5 days. This led to marked and rapid improvement with resolution of the serous detachment. The patient was discharged on an oral prednisone taper starting with 60 mg daily. Additionally, after reviewing available treatment options with the patient’s family, the rheumatology department recommended Infliximab therapy. Accordingly, the patient received Infliximab (750 mg/m2) infusions, with last dated treatment given six months following the initial hospitalization.

**Fig. 3:**
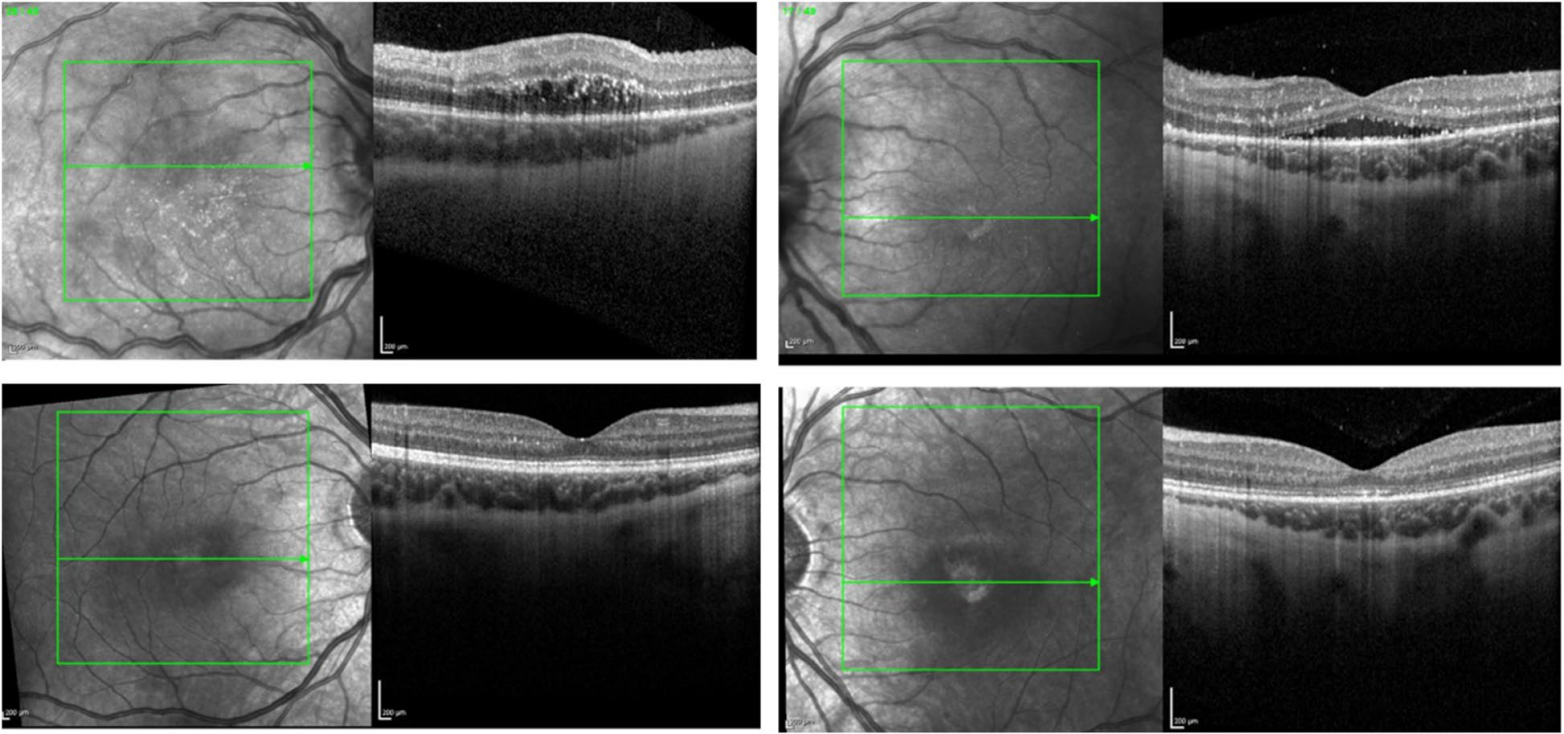
**A**, **B** Spectrum domain optical coherence tomography-macula (SD OCT-m) of the right and left eyes, taken a month after presentation demonstrates macular edema with intraretinal hyperreflective foci and disruption of the external limiting membrane, IS/OS junction and RPE of the right eye (3**A**) and intraretinal hyperreflective foci, subretinal fluid and hyperreflective foci at the level of the photoreceptors and RPE of the left eye (3**B**). **C**, **D** SD OCT-m of the right and left eyes taken at 4 month follow-up that demonstrates improved edema with few persistent intraretinal hyperreflective foci of the right eye (3C) and resolving subretinal fluid with improvement of the IS/OS junction in the left eye

On the one month outpatient follow up, visual acuity at distance was 20/300 OU. On slit lamp examination, she demonstrated trace cell in the anterior chamber with 1+ vitreous cell bilaterally. Indirect ophthalmoscope examination demonstrated residual, nasal optic nerve edema, resolution of peripheral vascular sheathing, and 360 chorioretinal scarring. Baseline optical coherence tomography demonstrated diffuse outer retinal layer disruption with edema superior to the macula OD and residual subretinal fluid OS (Fig. 3A and B).

Two weeks later, the visual acuity improved to 20/80 -1 OD and 20/150 OS. Exam showed resolving inflammation in her anterior chamber with a stable posterior examination. Repeat OCT imaging demonstrated persistent outer retinal layer disruption with resolved intraretinal edema OD and resolved subretinal fluid OS (Fig. 3c and d). Oral fluorescein angiography testing demonstrated late staining along the veins of the superior arcade with temporal perivascular leakage OD and late staining of venules superiorly with peripheral vascular dropout and perivascular leakage OS.

**Fig. 4:**
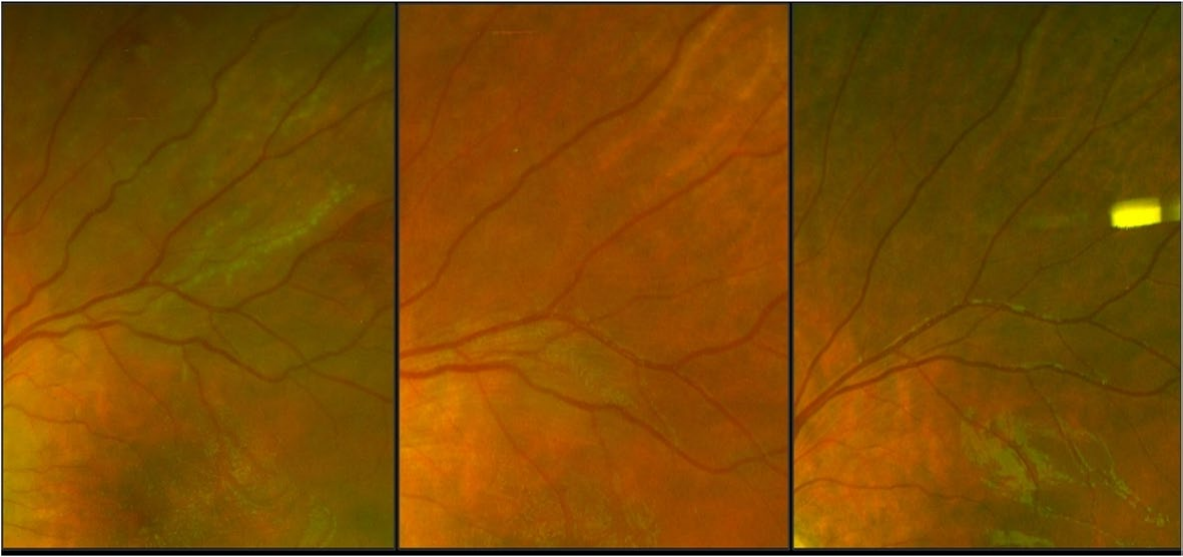
**A**, **B** and **C** Pseudo color wide-field fundus photographs of the left eye taken (4**a**) one month after presentation showing resolving serous detachment, vascular tortuosity and macular edema, and 4**b** six weeks after presentation. 4**c** taken at 4 months demonstrates return to relatively normal appearing vasculature with resolving of previously noted vascular sheathing

On 9 month clinical follow up, the patient’s visual acuity was 20/25-1 OD 20/30 OS. The examination demonstrated no active inflammation (Fig. 4).

## Discussion

Our case highlights important aspects of managing frosted branch angiitis. To begin, it requires interdisciplinary care between rheumatology, ophthalmology, hematology/oncology, infectious diseases, nephrology, and neurosurgery. Prior to ophthalmology consultation, the patient’s history and examination were suspicious for meningitis. After assessment by ophthalmology, a more targeted differential was formed and helped to guide management.

Furthemore, there are two significant aspects specific to this patient’s presentation. First, our case represents a unique ocular manifestation of COVID-19. Among patients diagnosed with COVID-19, the most common ophthalmic symptoms are dry eye, blurred vision, and foreign-body sensation [11]. There are reports of more serious involvement related to COVID-19, such as cases of unilateral retinal vasculitis and retinal vein occlusion [12–14]. However, the literature currently has only one reported COVI9-19 linked case of frosted angiitis, which occurred in an immunocompromised patient with positive CMV serologies [15].

Our case report adds to the literature, especially as the extensive laboratory workup had been overwhelmingly negative besides positive IgG antibodies to COVID 19 and a recently suspected infection. In this case, recent infection with SARS-CoV-2 was the only identifiable trigger for the patient’s frosted branch angiitis.

Finally, this case of frosted angiitis was refractory to intravenous corticosteroid therapy and required plasmapheresis to halt retinal progression. This is evidenced by the patient receiving IV corticosteroids for seven days without any improvement in their symptoms. There were three days of overlap, where the patient received combination of plasmapheresis and steroids. Likewise, Infliximab and Prednisone were administered as outpatient treatments. However, the initiation of plasmapheresis led to quick resolution that was noted prior to discharge. The literature does not demonstrate cases in which plasmapheresis was necessary for resolution of frosted angiitis. Though, apheresis therapy and plasmapheresis have been cited for other auto-immune related retinal vasculitis [16, 17].

## Conclusions

Prior infection with SARS-CoV-2 may represent an important cause of frosted-branch angiitis. The clinician should include questioning and testing to evaluate the possibility of recent infection when investigating this cause of retinal vasculitis. Lastly, when intravenous corticosteroids are failing to improve symptoms, escalation to plasmapheresis may be necessary to achieve resolution of symptoms.

## Data Availability

All data generated or analyzed during this study are included in this article. Further enquiries can be directed to the corresponding author.

## Abbreviations

HSV: Herpes Simplex Virus
VZV: Varicella Zoster Virus
AIDS: COVID-19, Acquired Immunodeficiency Syndrome
AC: Anterior Chamber
OCT: Ocular Coherence Tomography
ACE: Angiotensin-Converting Enzyme
